# Nonalcoholic fatty liver disease with elevated alanine aminotransferase levels is negatively associated with bone mineral density: Cross-sectional study in U.S. adults

**DOI:** 10.1371/journal.pone.0197900

**Published:** 2018-06-13

**Authors:** Toshihiro Umehara

**Affiliations:** Department of Epidemiology, Rollins School of Public Health, Emory University, Atlanta, Georgia, United States of America; Universita degli Studi di Verona, ITALY

## Abstract

**Background:**

Nonalcoholic fatty liver disease (NAFLD) has been reported to have a negative effect on bone mineral density (BMD) in Asian populations. Whether such an association exists in Western populations is less clear.

**Methods:**

This cross-sectional analysis of data from NHANES III, a United States national health survey conducted from 1988 to 1994, included 6089 participants aged 40–75 years, selected after excluding people with hepatitis virus serology, elevated alcohol consumption, decreased renal function, or steroid use, and pregnant females. The main outcome, BMD at the femoral neck, was measured using dual-energy X-ray absorptiometry. The primary exposure, NAFLD, was defined as moderate or severe hepatic steatosis diagnosed using abdominal ultrasonography.

**Result:**

After controlling for gender and menopausal status, race/ethnicity, age and body mass index, NAFLD was not significantly associated with BMD (beta coefficient: −0.006, 95%CI: −0.016, 0.003). A secondary analysis categorized participants with NAFLD according to their serum alanine aminotransferase (ALT) levels into high and normal ALT NAFLD groups, and compared these with the non-NAFLD group. NAFLD with higher levels of ALT was associated with lower levels of BMD (beta coefficient: −0.023, 95% CI: −0.044, −0.002).

**Conclusion:**

This study showed a relationship between NAFLD with high ALT and lower BMD in the general U.S. population.

## Introduction

Nonalcoholic fatty liver disease (NAFLD) is characterized by an excessive intrahepatic fat deposition without a specific cause of secondary hepatic steatosis, such as excessive alcohol consumption, viral hepatitis, or a hereditary disorder [1, 2]. A high-calorie diet and a sedentary lifestyle contribute to the NAFLD development [3]. NAFLD is the most prevalent chronic liver disease in Western countries, with a prevalence in the general population of 25%-45% [4], and its incidence is increasing in parallel with the increase in obesity [5].

Most cases of NAFLD involve patients with a metabolically abnormal condition, such as obesity, diabetes mellitus, or dyslipidemia [2]. For this reason, previously NAFLD was thought to be a liver manifestation of metabolic syndrome (MetS) and insulin resistance. However, there is increasing evidence of interaction between NAFLD and MetS or insulin resistance [1]. NAFLD does not only occur in patients with MetS, but also NAFLD can occur in lean individuals [6, 7], and it can be a precursor to MetS and diabetes mellitus [8, 9].

As well as MetS and insulin resistance, multiple factors and organs interact with NAFLD, resulting in the development a systemic condition [3]. The risk of cardiovascular disease is reported to be higher among patients with NAFLD than among those without NAFLD, independently of obesity and other atherosclerosis risk factors [10]. Studies have shown associations between NAFLD and subclinical myocardial remodeling and dysfunction [11], arrhythmia [12], and early carotid atherosclerosis [13]. It has also been reported to be associated with an increased risk of some types of cancers, including hepatocellular carcinoma, colorectal cancer and breast cancer [14, 15]. The development of chronic kidney disease also shows an association with NAFLD [16]. These findings indicate that NAFLD is a systemic disease, not limited to the liver.

Similarly, a relationship between NAFLD and osteoporosis has been suggested. Osteoporosis is “characterized by low bone mass, the deterioration of bone tissue, and disruption of bone architecture, compromised bone strength and an increase in the risk of fracture.”, which is diagnosed by measuring bone mineral density (BMD) [17]. A meta-analysis has shown that, after adjusting for covariates, groups of people with MetS have lower BMDs than control groups [18]. MetS and NAFLD share many characteristics and causes, so a similar relationship could exist between NAFLD and BMD.

Epidemiological studies in Asia have demonstrated an association between NAFLD and low BMD [19–21] as well as a higher risk of osteoporotic fracture in people with NAFLD [22]. Moon et al. reported the association among postmenopausal women in Korea [19]. They found a significant association between lumbar BMD and NAFLD, even after adjusting for age, body mass index, serum alanine aminotransferase (ALT), smoking status, and alcohol consumption (β coefficient −0.066, 95%CI: −0.105 to −0.027). Cui et al. also reported associations between NAFLD and lower BMD in men and postmenopausal women in China [20]; this association was observed after adjusting for weight, body mass index (BMI), waist size, high density lipoprotein (HDL) concentration, and ALT, but it became non-significant after further adjusting for insulin resistance (HOMA-IR), suggesting an important role of insulin resistance in the NALFD-BMD association.

In the present study, participants with NAFLD were categorized according to their serum ALT levels. ALT is a liver injury biomarker routinely measured in clinical settings, and it has been reported to be related to systemic metabolic conditions. Increased levels of ALT are associated with the long-term development of multiple metabolic diseases, including MetS and diabetes mellitus [23]. Martin-Rodriguez et al. showed correlation of ALT with liver fat content and increased insulin resistance [24], suggesting that ALT may be useful for NAFLD categorization when evaluating NAFLD’s systemic relationships.

In this study, we used U.S. national health and nutrition survey data to assess the relationship between NAFLD and BMD for the general population. Such a relationship has not been fully examined especially in Western countries. The main research question was whether NAFLD showed a negative relationship with BMD, and the secondary research question was how ALT levels affected this relationship. The result of this study should provide greater insight into NAFLD, and should be beneficial for future research and preventive medicine for patients with NAFLD.

## Methods

### Study population and design

This cross-sectional analysis used data from the U.S. National Health and Nutrition Survey (NHANES) III [25]. NHANES is an ongoing national survey designed to assess the health and nutritional status of adults and children in the United States. The surveys collect data about the participants’ health and nutritional status through interviews and laboratory tests, as well as demographic and socioeconomic status. This survey program began in the early 1960s; NHANES III (from 1988 to 1994) was the seventh in this series of surveys. We used NHANES III data for the present study because it was the only survey with one that has available liver ultrasonography measures.

NHANES III was designed to represent the U.S. civilian, non-institutionalized population through complex, multi-stage sampling. The designated total sample size was 39,695. Among them, 30,818 were examined at the mobile examination centers (MEC). In this study, we initially included all the participants from 40–75 years old (3817 males and 4131 females). We then excluded participants from the analysis based on the following criteria. 1) People with hepatitis B e antigen (HBeAg) positive or hepatitis C virus antibody positive (n = 202); 2) Elevated alcohol consumption, defined by men drinking more than 2 drinks per day or women drinking more than 1 drink per day [26] (n = 535); 3) Decreased renal function, defined by an estimated glomerular filtration rate (eGFR) less than 30 (ml/min/1.73m^2^) according to the Modification of Diet in Renal Disease (MDRD) study equation (n = 37). MDRD equation: eGFR = 175 × (standardized serum creatinine)^-1.154^ × (age)^-0.203^ (× 0.742 if the subject is female) (×1.212 if the subject is black). The equation requires serum creatinine level [27], for which the original value in NHANES III needs to be calibrated [28]. Calibration for serum creatinine (mg/dl): Standardized Serum Creatinine = 0.960*NHANES Creatinine– 0.184; 4) People with thyroid stimulating hormone (TSH) lower than 0.1 mU/L: People suspected of hyperthyroidism which can accelerate the decrease in BMD and have an influence on MetS were excluded (n = 0); 5) Patients taking prednisone, prednisolone, methylprednisolone, betamethasone, dexamethasone or triamcinolone for >90 days (n = 76); 6) Pregnant females (n = 2).

**Fig 1:**
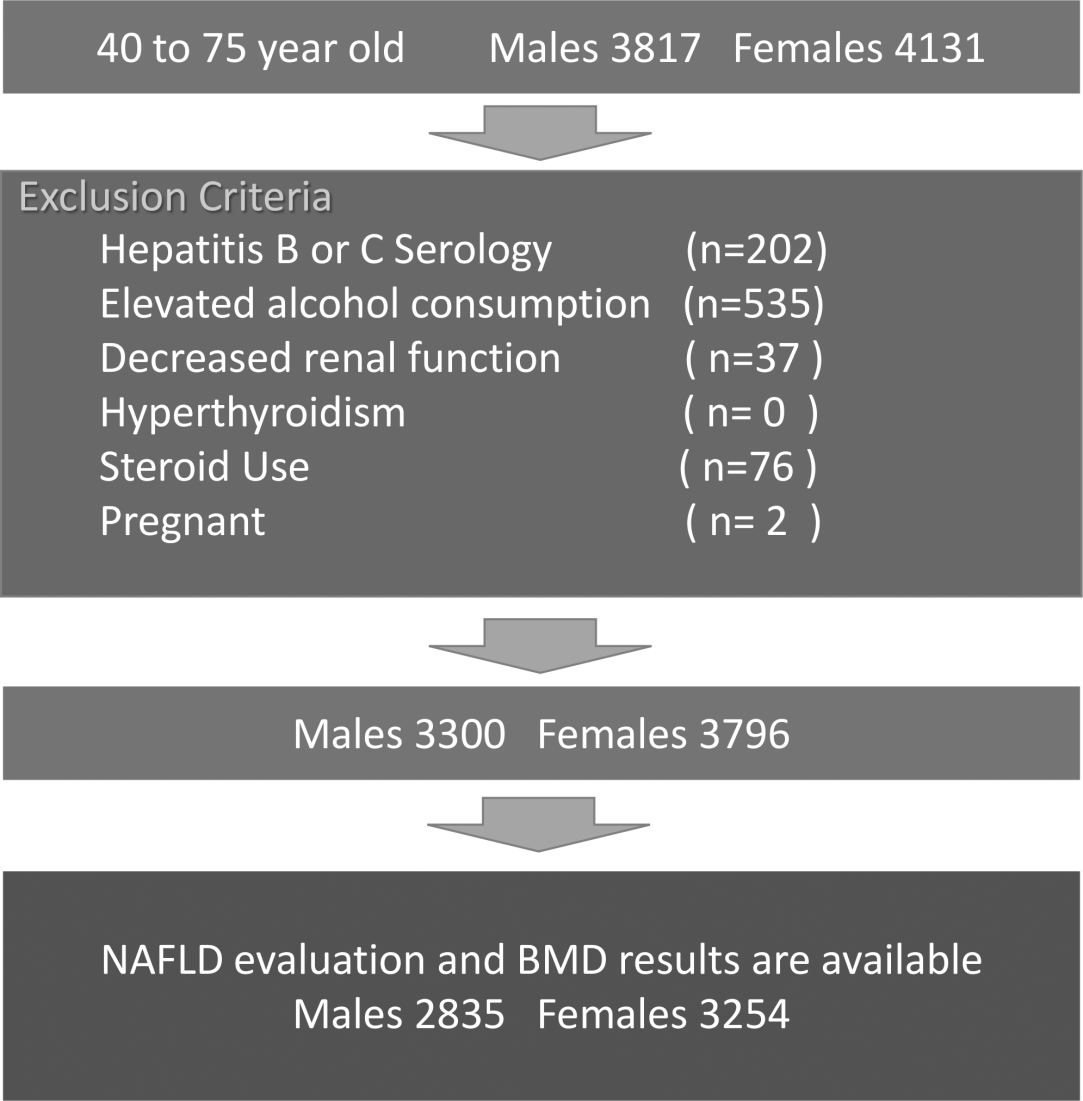
Participant selection. In the final step, 267 males and 266 females were excluded because of the lack of available ultrasonography results. 198 males and 276 females were excluded because bone mineral density results were not available.

Following the exclusions, a total of 3300 males and 3796 females remained. Among these people, ultrasonography results were unavailable for 267 males and 266 females, and BMD results were unavailable for 198 males and 276 females. Finally, 2835 males and 3254 females (6089 participants overall) remained, and they were used in the analysis (Fig 1).

### Measurements

#### Bone mineral density

BMD at the femoral neck was used as the primary outcome because it is highly predictive of hip fractures [29, 30]. BMD was measured using dual-energy X-ray absorptiometry (DEXA) instruments (QDR-1000, Hologic, Waltham, MA). A quality control program was used to ensure data quality [31]. The examination was not performed for females with a positive or uncertain pregnancy test, females with the possibility of pregnancy, people whose hips had both been fractured or broken previously, and people with hip pins or artificial hips [32].

#### Definition of NAFLD

In this study, NAFLD was identified by a diagnosis of liver steatosis on abdominal ultrasonography in participants without a competing etiology for secondary liver steatosis, such as excessive alcohol intake, positive viral hepatitis serology, steroid use, or abnormal thyroid hormone levels. In NHANES III, the degree of liver steatosis was recorded as either a normal liver or severe, moderate, or mild steatosis, based on the following parameters [26]: "1) the presence of liver-to-kidney contrast (yes, no, or not assessed); 2) the degree of brightness of the liver parenchyma (none, intermediate, moderate, or severe); 3) the presence of posterior deep beam attenuation (yes, no, or not assessed); 4) the presence of echogenic walls in the small intrahepatic vessels (yes, no, or not assessed); and 5) the definition of the gallbladder walls (clear, intermediate, obliterated, or not assessed)." The classification was conducted in a standardized way; a detailed description of this protocol has been reported elsewhere [33].

The models of this study assessed the effect of NAFLD as the main exposure. In the primary analysis, NAFLD was treated as a dichotomous variable, that is as present (with moderate or severe steatosis) or absent (normal liver or with mild steatosis) [26]. In the secondary analysis, the participants with NAFLD were categorized into two groups according to their ALT levels: high ALT and normal ALT (the HA NAFLD and NA NAFLD groups, respectively). Various studies have proposed normal ranges for ALT levels [34–37]. In this study, we used the ranges reported by Ruhl and Everhart, for which the upper limits were 29 U/L for men and 22 U/L for women [36]. These ranges were based on large samples representative of the U.S. population (the NHANES 1999–2002 and 2005–2008 surveys), and so were suitable for use in our study. The participants without NAFLD were categorized into non-NAFLD group.

#### Menopausal status

Menopausal status was defined according to previously reported coding scheme [38, 39]. The following criteria were applied sequentially so that each successive rule was applied only to women not already categorized: a) age ≥ 60 years, postmenopausal; b) bilateral oophorectomy, postmenopausal; c) a period or pregnancy within the previous 12 months, premenopausal; d) follicle-stimulating hormone level > 40 IU/L, postmenopausal; e) current use of oral contraceptive pill, premenopausal; and f) no menstrual period experience during the previous year, postmenopausal. After this categorization, the menopausal status of 39 females remained unclear; of these, 11 females aged >50 years were considered postmenopausal, and the other 28 were considered premenopausal.

#### Osteoporosis and osteopenia

Osteoporosis and osteopenia were defined as having BMD at the femoral neck of ≤0.56 g/cm2 and ≤0.74 g/cm2 respectively [40].

### Statistical analysis

**Fig 2:**
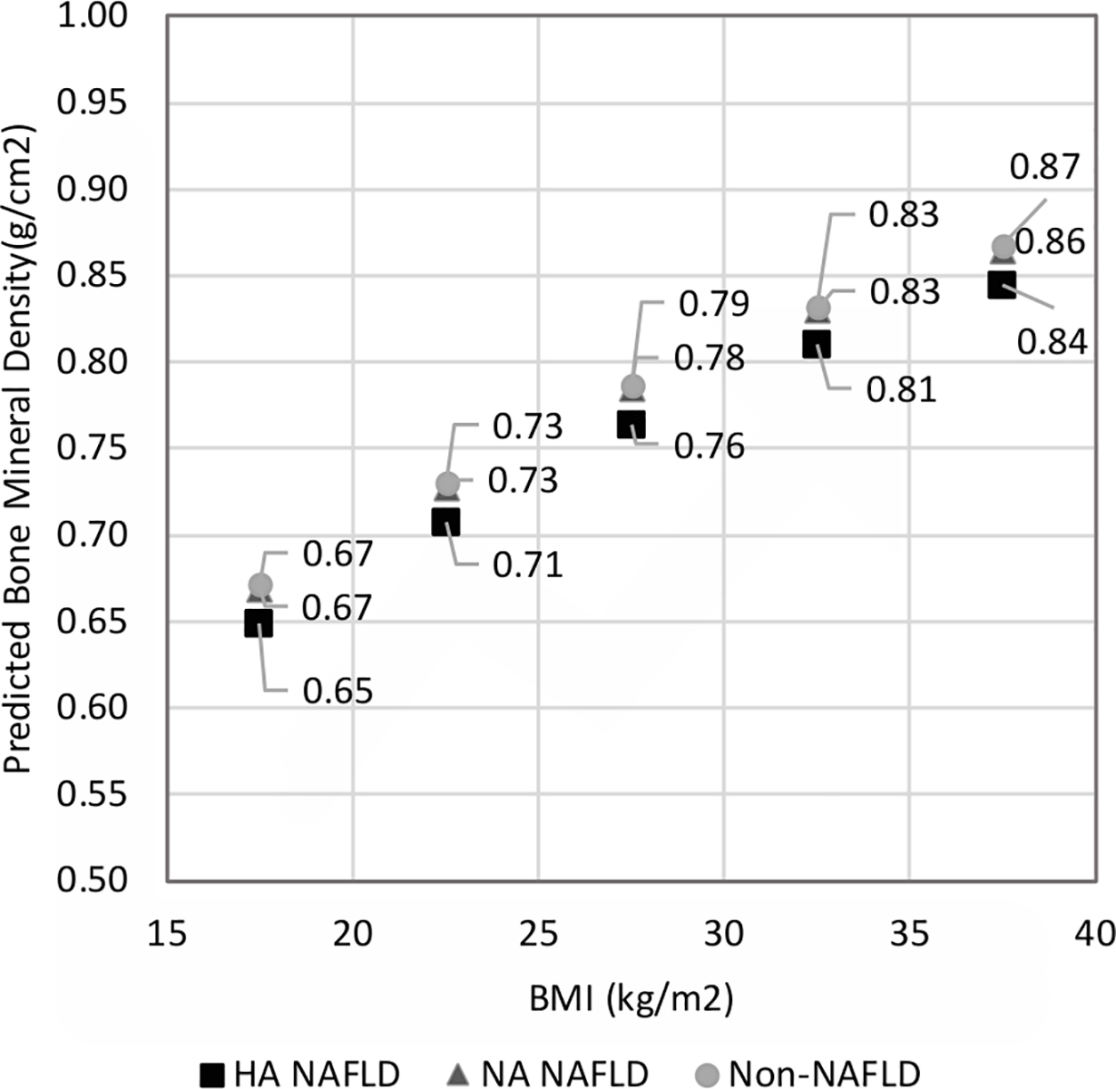
Predicted femoral neck bone mineral densities for HA NAFLD, NA NAFLD and non-NAFLD groups for different levels of BMI. The predictions were made using the final model, Model 2. Representative values were used for covariates for each group (described in detail in method section). Abbreviations: HA NAFLD, NAFLD with high alanine aminotransferase levels; NA NAFLD, NAFLD with normal alanine aminotransferase levels.

NHANES III was conducted based on complex, multi-stage sampling; in this study we took account of its survey design and sampling weights [41, 42]. All the analyses were performed using SAS-Callable SUDAAN Ver. 11.0. Subpopulations were specified by using SUBPOPN or SUBPOPX statements to prevent losing design effect information. In the descriptive analyses, statistics such as means and proportions were calculated using PROC DESCRIPT. Multiple linear regression analysis was performed using PROC REGRESS. The key covariates and variables for tests of interaction were selected based on previous reports (S1 Fig). Participants who were categorized into other race/ethnicities (than non-Hispanic White, non-Hispanic Black and Mexican-American) included other Hispanics, Asians, and Native Americans. Those with other race/ethnicities were included in the overall descriptive analyses, but were not included in the regression analyses, because the group’s sample size was too small to be used analytically and the category was too difficult to label [42]. The dataset was first fitted with the full models that included the exposure variable (NAFLD in the primary analysis and HA or NA NAFLD in the secondary analysis), confounders (gender and menopausal status, race/ethnicity, age groups, BMI), and all the interaction terms between the exposure variable and confounders. Starting with the full model, the interaction term with least statistical significance was iteratively removed using backward elimination until there were no insignificant interaction terms or there were no remaining interaction terms (S2 and S6 Tables). In this way, the final models, Model 1 and Model 2, were obtained from the primary analysis and the secondary analysis, respectively. Fig 2 shows the femoral neck BMDs predicted for the different NAFLD groups for different BMI ranges using the final model (Model 2) obtained from the secondary analysis. The predictions were calculated after stratifying the population into BMI rages of 15–20, 20–25, 25–30, 30–35, and 35–40 kg/m^2^. When calculating the predicted BMDs, representative values to be entered as covariates for Model 2 were chosen from each NAFLD and BMD group as follows: for the age covariate, the mean age for each group; for BMI, the midpoint of each range (e.g. 22.5 kg/m^2^ was used for the 20–25 kg/m^2^ BMI group); and for categorical variables, gender and menopausal status and race/ethnicity, the proportion of each group.

## Results

Overall 25% of participants had moderate or severe NAFLD (Table 1). The prevalence of moderate or severe NAFLD was higher in men (28.2%) than in women (22.1%), and higher in postmenopausal females (25.0%) than in premenopausal females (16.4%). Participants in the NAFLD group were also older and heavier than those in the non-NAFLD group (Table 1). Femoral neck BMD was higher in the NAFLD group, and unadjusted analysis of the NAFLD effect showed a statistically significant protective effect on BMD (beta coefficient: +0.032, 95% confidence intervals (CI): 0.021, 0.042). The proportions of participants with osteopenia and osteoporosis were lower in the NAFLD group, though these results may have been affected by the larger BMI and body weight of the participants in this group (Table 1).

**Table 1:** Characteristics of participants with and without NAFLD (N = 6089).

	NAFLD	Non-NAFLD	P value
	n = 1690	n = 4399	
Sum of Sample Weights (%)	24.9	75.1	
Demographic variables			
Gender (%)			< 0.01
Males	52.8	44.8	
Premenopausal females	11.8	19.9	
Postmenopausal females	35.4	35.3	
Race/Ethnicity (%)			< 0.01
White	80.5	80.0	
Black	7.4	9.6	
Mexican-American	5.8	3.0	
Others	6.3	7.4	
Age (years)	55.6	54.0	< 0.01
Body Weight (kg)^1^	87.6	74.3	< 0.01
BMI (kg/m2)^1^	30.7	26.4	< 0.01
Clinical variables			
AST (U/L)^2^	23.5	20.0	< 0.01
ALT (U/L)^2^	22.2	15.2	< 0.01
Bone mineral density			
Femoral neck (g/cm2)	0.80	0.77	< 0.01
Osteoporosis (%)	3.6	5.6	0.01
Osteopenia (%)	33.4	42.8	< 0.01

Abbreviations: AST, aspartate aminotransferase; ALT, alanine aminotransferase.

Values are presented as the weighted mean or weighted percentage. The NAFLD group included the participants with moderate or severe steatosis, and the non-NAFLD group included those with normal livers or mild steatosis.

^1^ N = 6079

^2^ N = 5800

When the dataset was stratified by gender and menopausal status, race/ethnicity or age ranges, the NAFLD group showed higher BMD values than the non-NAFLD group (Table 2). In contrast, when the dataset was stratified by BMI ranges, the NAFLD group showed lower BMD values than the non-NAFLD group, although these differences were not statistically significant (Table 2).

**Table 2:** Femoral neck bone mineral densities for participants with and without NAFLD, stratified by gender and menopausal status, race/ethnicity, age, and BMI.

	NAFLD	Non-NAFLD	P value
Gender and menopausal status			
Males	0.839 (0.007)	0.813 (0.004)	< 0.01
Premenopausal Females	0.827 (0.010)	0.804 (0.007)	0.06
Postmenopausal Females	0.741 (0.007)	0.701 (0.005)	< 0.01
Race/Ethnicity			
White	0.793 (0.005)	0.760 (0.004)	< 0.01
Black	0.900 (0.010)	0.868 (0.006)	< 0.01
Mexican American	0.832 (0.006)	0.813 (0.005)	0.01
Age			
40–50 years	0.863 (0.008)	0.819 (0.005)	< 0.01
50–60 years	0.801 (0.009)	0.771 (0.006)	< 0.01
60–75 years	0.754 (0.007)	0.712 (0.004)	< 0.01
BMI			
- 25 kg/cm2	0.712 (0.012)	0.725 (0.005)	0.32
25–30 kg/cm2	0.781 (0.008)	0.788 (0.004)	0.35
30–35 kg/cm2	0.822 (0.008)	0.827 (0.009)	0.63
35—kg /cm2	0.875 (0.010)	0.878 (0.013)	0.81

Data are expressed as estimates (standard error). The NAFLD group included the participants with moderate or severe steatosis, and the non-NAFLD group included those with normal livers or mild steatosis.

For the primary objective of this study, adjusted effects of NAFLD on BMD were assessed using multiple linear regression, controlling for gender and menopausal status, race/ethnicity, age, and BMI, as well as their interaction terms with NAFLD (S2 Table). Although the final model (Model 1 in Table 3) showed a small negative effect of NAFLD on BMD (beta coefficient: −0.006, 95%CI: −0.016, 0.003), this was not statistically significant.

**Table 3:** Multiple linear regression analysis of the effect of NAFLD on bone mineral density (N = 5822).

	Model 1	P value
Intercept	0.812 (0.004)	< 0.01
NAFLD	−0.006 (0.005)	0.19
Non-NAFLD	Ref.	
Postmenopausal Female	−0.100 (0.005)	< 0.01
Premenopausal Female	−0.039 (0.006)	< 0.01
Male	Ref.	
Black	0.090 (0.005)	< 0.01
Mexican-American	0.025 (0.004)	< 0.01
White	Ref.	
Age	−0.0034 (0.0002)	< 0.01
BMI	0.0096 (0.0005)	< 0.01

Data are expressed as beta estimates (standard error). The NAFLD group included the participants with moderate or severe steatosis, and the non-NAFLD group included those with normal livers or mild steatosis. Only Black, Mexican-American, and White participants were used in this analysis, and participants with other race/ethnicities were not used (described in method section). Age and BMI were dealt as continuous variables. All the interaction terms between NAFLD and the other covariates were included in the primary full model, and statistically insignificant interaction terms were removed iteratively using backward elimination (described in detail in S2 Table). Finally, the final model (Model 1) did not include any interaction terms.

In the secondary analysis, we further categorized the NAFLD group into two groups, NAFLD with high ALT (HA NAFLD) and NAFLD with normal ALT (NA NAFLD), and compared them with non-NAFLD group. In the final model (Model 2 in Table 4), the main effect of HA NAFLD showed a statistically significant negative effect on BMD (beta coefficient: −0.023, 95% CI: −0.044, −0.002). Predicted BMDs using the final model, Model 2, were illustrated in Fig 2.

**Table 4:** Secondary multiple linear regression analysis assessing the effects of NAFLD with high or normal alanine aminotransferase (ALT) levels on bone mineral density (N = 5751).

	Model 2	P value
Intercept	0.812 (0.004)	< 0.01
High ALT (HA) NAFLD	−0.0230 (0.0104)	0.03
Normal ALT (NA) NAFLD	−0.0027 (0.0053)	0.62
Non-NAFLD	Ref.	
Postmenopausal	−0.100 (0.005)	< 0.01
Premenopausal	−0.039 (0.006)	< 0.01
Male	Ref.	
Black Mexican-American White	0.089 (0.005) 0.026 (0.004) Ref.	< 0.01 < 0.01
Age	−0.0034 (0.0002)	< 0.01
BMI	0.0097 (0.0005)	< 0.01

Data are expressed as beta estimates (standard error). The HA NAFLD group included participants with moderate or severe steatosis with high ALT levels, the NA NAFLD group included participants with moderate or severe steatosis with normal ALT levels, and the non-NAFLD group included participants with mild steatosis or normal liver. Only Black, Mexican-American, and White participants were used in this analysis, and participants with other race/ethnicities were not used (described in method section). Age and BMI were dealt as continuous variables. Model 2 was the final model derived from the full model in the secondary analysis. Interaction terms in the full model were assessed, and statistically insignificant terms were removed iteratively using backward elimination (described in detail in S6 Table). Finally, the final model (Model 2) included no interaction terms. Abbreviations: HA NAFLD, NAFLD with high alanine aminotransferase levels; NA NAFLD, NAFLD with normal alanine aminotransferase levels.

## Discussion

The primary analysis in this study did not show a statistically significant association between nonalcoholic fatty liver disease (NAFLD) and bone mineral density (BMD) (Table 3). However, in the secondary analysis, which categorized NAFLD according to ALT levels, showed a statistically significant negative effect of high ALT NAFLD on BMD (Table 4).

**Fig 3:**
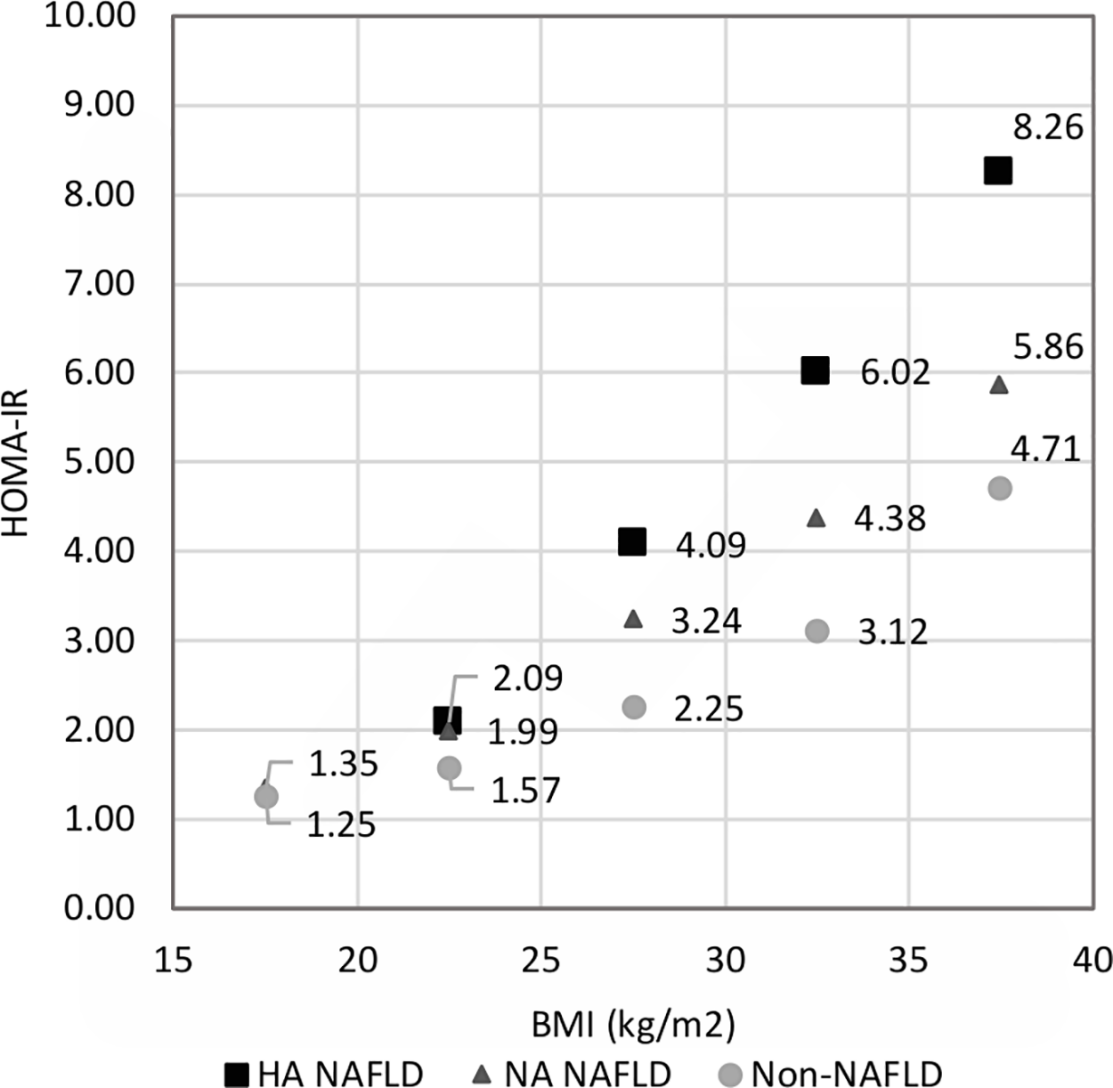
HOMA-IR levels for the different NAFLD groups for different BMI ranges. The population was stratified by BMI into the ranges 15–20, 20–25, 25–30, 30–35, and 35–40 kg/m2. No one in the HA NAFLD group had a BMI in the range 15–20 kg/m2. Mean HOMA-IR values were obtained for each NAFLD group for the different BMI ranges. Abbreviations: HOMA-IR, homeostatic model assessment of insulin resistance; HA NAFLD, NAFLD with high alanine aminotransferase levels; NA NAFLD, NAFLD with normal alanine aminotransferase levels.

The result of the secondary analysis may be partly explained by some systemic condition that is related to higher levels of ALT. Maximos et al. assessed underlying factors of plasma ALT elevations in patients with biopsy-proven NAFLD/NASH, and showed that adipose tissue insulin resistance and liver triglyceride content are majors factors for elevated plasma aminotransferase levels [43]. Martin-Rodriguez et al. reported the statistically significant correlation of serum ALT with insulin resistance and liver fat content [24]. Goessling et al. showed the association between ALT levels and the development of incidents of diabetes over 20 year of follow-up [23], and Schindhelm et al. reported the association between ALT levels and coronary heart disease events [44]. Similarly, in this study, the HA NAFLD group showed stronger insulin resistance than the NA NAFLD and non-NAFLD groups (Fig 3, S8 Table), a finding consistent with the previous reports. All these results support that ALT levels are related to some metabolically abnormal systemic condition, and it may have contributed to the negative effect of high ALT NAFLD on BMD in the secondary analysis of this study.

To further investigate the underlying mechanism of the relationship between HA NAFLD and BMD, multiple liner regression analysis was performed to assess the effects of HA and NA NAFLD on BMD for males, postmenopausal females, and premenopausal females (S7A–S7C Table). A statistically significant negative effect of HA NAFLD on BMD was observed only for males (Model 2A in S7A Table). This model also had a positive interaction term between HA NAFLD and BMI, indicating that the main negative effect of HA NAFLD on BMD was attenuated as BMI increased and strengthened as BMI decreased. S2 Fig illustrates how the predicted BMD changed with BMI for the different ALT/NAFLD groups among males. This analysis stratified by gender and menopausal status showed that the effect of HA NAFLD on BMD could vary with BMI and gender. These findings may also explain why the effect of NAFLD on BMD in the primary analysis did not achieve statistical significance, in contrast to the results in previous reports from Asia [19–21]. Not only race/ethnicities, but also the distributions of ALT, BMI and gender in the sample population may have influenced the primary result.

Vitamin D levels may also explain the varying effects of HA NAFLD on BMD among males. Relationships between vitamin D levels and NAFLD and BMD have been reported [45–49]. When 25 hydroxyvitamin D levels (25(OH) D) were compared between the NAFLD groups and BMI ranges in males (S3 Fig, S11 Table), the HA NAFLD group showed lower 25(OH) D levels than the NA NAFLD and non-NAFLD groups among those with BMI of 20–30. However, the relationship reversed among those with BMI >30. Thus, HA NAFLD males with BMI of 20–30 may fail to receive the protective effect on BMD from 25(OH) D, whereas vitamin D levels may attenuate negative relationship between HA NAFLD and BMD in men with higher BMI. However, these inferences are based on a descriptive analysis and further research is required to assess the importance of these factors.

A strength of this analysis was the generalizability of our study population, as the study sampled several race/ethnic groups, including Whites, Blacks, and Mexican Americans. Another strength was the validity of the coefficients for covariates in the final models, which justified the models and their appropriateness to test the main effect of NAFLD (Tables 3 and 4). The variables such as gender and menopausal status, race/ethnicity, age, and BMI showed statistically significant effects consistent with previous biological and epidemiological explanations. Females have lower BMD than males, and menopause accelerates the decrease in BMD [17]. Non-Hispanic blacks have higher BMD than white people [50, 51], and as people age, BMD decreases. BMI and body size have a protective effect against BMD reduction [17].

This study also had some limitations. First, it was a cross-sectional analysis, which leaves open the possibility for reverse causality. Second, people taking corticosteroids were excluded in this study, but information of other drugs was not used. There are some other drugs that can potentially modify NAFLD-BMD relationship. Third, it may be argued that including people with a history of bone fracture or with a history of osteoporosis treatment may affect the NAFLD-BMD relationship, though the direction of bias is unclear. Sensitivity analysis was conducted to address this concern. The final model was fit to the dataset excluding those people who answered they had experienced a bone fracture of the hip, wrist or spine, or who answered they had received osteoporosis treatment, and the effects of HA NAFLD and NA NAFLD were reassessed. The main effect of HA NAFLD showed a statistically significant negative effect on BMD (beta coefficient: −0.025, 95%CI: −0.049, −0.001); this result was consistent with that of the main analysis. Fourth, ALT was used to categorize participants with NAFLD, but this categorization does not directly represent the severity of NAFLD or the degree of fibrosis. Rather, as mentioned above, ALT could represent some metabolically abnormal systemic condition. Thus, caution is needed when interpreting the result. Non-invasive hepatic fibrosis markers, such as Fib-4 score and NAFLD fibrosis score (NFS), have been developed to evaluate the severity of hepatic diseases without a liver biopsy [52–54], and these markers have been shown to be useful for evaluating NAFLD and its prognosis [55]. Future studies to assess the relationships between these hepatic fibrosis markers and BMD would be beneficial.

In conclusion, this study showed a statistically significant relationship between high ALT NAFLD and lower BMD in the general U.S. population. Serum ALT levels may be related to an abnormal systemic metabolic status in people with NAFLD. These results could be beneficial for future research and for preventive medicine for patients with NAFLD.

## Supporting information

S1 Table Detailed characteristics of participants of this study. (DOCX)

S2 Table Multiple linear regression analysis of the effect of NAFLD on bone mineral density. (DOCX)

S3 Table Multiple linear regression analysis of the effect of NAFLD on bone mineral density. (A) Males (B) Premenopausal Females (C) Postmenopausal Females.
(DOCX)

S4 Table Characteristics of participants with NAFLD with high or normal ALT levels and participants without NAFLD. (DOCX)

S5 Table Femoral neck BMD for participants with NAFLD with high or normal alanine aminotransferase (ALT) levels and participants without NAFLD, stratified by gender and menopausal status, race/ethnicity, age, and BMI. (DOCX)

S6 Table Secondary multiple linear regression analysis assessing the effects of NAFLD with high or normal alanine aminotransferase (ALT) levels on bone mineral density. (DOCX)

S7 Table Secondary multiple linear regression analysis assessing the effects of NAFLD with high or normal alanine aminotransferase (ALT) levels on bone mineral density for each gender and menopausal status (A) Males (B) Premenopausal Females (C) Postmenopausal Females.
(DOCX)

S8 Table Mean values of HOMA-IR for the NAFLD groups for different levels of BMI. (DOCX)

S9 Table Mean values of HOMA-IR for the NAFLD groups for different levels of BMI among males. (DOCX)

S10 Table Mean values of serum vitamin D (25 OH D) for the NAFLD groups for different levels of BMI. (DOCX)

S11 Table Mean values of serum vitamin D (25 OH D) for the NAFLD groups for different levels of BMI among males. (DOCX)

S1 Fig The association assessed in this study is confounded by gender and menopausal status, age, BMI, and races. The association assessed in this study is confounded by gender and menopausal status, age, BMI, and races.
(DOCX)

S2 Fig Predicted femoral neck bone mineral densities for the NAFLD groups for different levels of BMI among males. (DOCX)

S3 Fig 25(OH)D levels for the NAFLD groups for different levels of BMI among males. (DOCX)

S1 File STROBE checklist cross-sectional. (DOC)

S2 File Source codes for this study. (ZIP)
