# Supplementary material for: Nonalcoholic fatty liver disease with elevated alanine aminotransferase levels is negatively associated with bone mineral density: Cross-sectional study in U.S. adults
Source: PLoS One. 2018 Jun 13;13(6):e0197900. doi: 10.1371/journal.pone.0197900 (PMC5999215; doi:10.1371/journal.pone.0197900)
Supplement: S1 Table — (DOCX) [file pone.0197900.s001.docx]

S1 Table. Detailed characteristics of participants of this study (n=6089)

|  | NAFLD | Non-NAFLD | P value |
| --- | --- | --- | --- |
| Creatinine (mg/dl) ^1^ | 0.87 | 0.85 | <0.01 |
| Estimated GFR (ml/min) ^1^ | 84.6 | 85.7 | 0.11 |
| Calcium (mmol/L) ^2^ | 1.23 | 1.23 | 0.49 |
| Thyroid Stimulating Hormone(TSH) (U/ml) ^3^ | 2.69 | 2.32 | 0.17 |
| Platelet (10^4/μL) ^4^ | 27.3 | 27.0 | 0.39 |
| 25(OH) Vitamin D (ng/ml) ^5^ | 69.6 | 72.0 | 0.14 |
| HOMA-IR ^6^ | 4.38 | 2.20 | <0.01 |

^1^ N=5800, ^2^ N=5360, ^3^ N=5732, ^4^ N=5884, ^5^ N=5917, ^6^ N=5008
