# Supplementary material for: Nonalcoholic fatty liver disease with elevated alanine aminotransferase levels is negatively associated with bone mineral density: Cross-sectional study in U.S. adults
Source: PLoS One. 2018 Jun 13;13(6):e0197900. doi: 10.1371/journal.pone.0197900 (PMC5999215; doi:10.1371/journal.pone.0197900)
Supplement: S2 Table — (DOCX) [file pone.0197900.s002.docx]

S2 Table. Multiple linear regression analysis of the effect of NAFLD on bone mineral density (N=5822)

|  | Full Model | P value | Model 1 | P value |
| --- | --- | --- | --- | --- |
| Intercept | 0.811 (0.004) | < 0.01 | 0.812 (0.004) | < 0.01 |
| NAFLD | −0.002 (0.008) | 0.84 | −0.006 (0.005) | 0.19 |
| Non-NAFLD | Ref. |  | Ref. |  |
| Postmenopausal Female | −0.098 (0.005) | < 0.01 | −0.100 (0.005) | < 0.01 |
| Premenopausal Female | −0.036 (0.007) | < 0.01 | −0.039 (0.006) | < 0.01 |
| Male | Ref. |  | Ref. |  |
| Black | 0.088 (0.005) | < 0.01 | 0.090 (0.005) | < 0.01 |
| Mexican-American | 0.027 (0.005) | < 0.01 | 0.025 (0.004) | < 0.01 |
| White | Ref. |  | Ref. |  |
| Age | −0.0034 (0.0002) | < 0.01 | −0.0034 (0.0002) | < 0.01 |
| BMI | 0.0098 (0.0007) | < 0.01 | 0.0096 (0.0005) | < 0.01 |
| Interaction terms |  |  |  |  |
| NAFLD*Gender/Menopausal |  | 0.67 |  |  |
| NAFLD*Postmenopausal | −0.007 (0.012) | 0.58 |  |  |
| NAFLD*Premenopausal | −0.013 (0.016) | 0.41 |  |  |
| NAFLD*Male | Ref. |  |  |  |
| NAFLD*Races |  | 0.51 |  |  |
| NAFLD*Black | 0.007 (0.010) | 0.47 |  |  |
| NAFLD*Mexican-American | −0.004 (0.008) | 0.64 |  |  |
| NAFLD*White | Ref. |  |  |  |
| NAFLD*Age | 0.0002 (0.0005) | 0.76 |  |  |
| NAFLD*BMI | −0.0006 (0.0010) | 0.56 |  |  |

Data are expressed as beta estimates (standard error). The NAFLD group included the participants with moderate or severe steatosis, and the non-NAFLD group included those with normal livers or mild steatosis. Only Black, Mexican-American, and White participants were used in this analysis, and participants with other race/ethnicities were not used (described in method section). Age and BMI were dealt as continuous variables. Age variable and BMI variable were centered in these models around overall means, 54 and 27 respectively.

Full model had gender and menopausal status, race/ethnicity, age, BMI, and their interactions with NAFLD status as covariates. Interaction terms in the full model were assessed, and statistically insignificant terms were removed iteratively using backward elimination. As a result, all the interaction terms were not included in the final model, Model 1.
