# Supplementary material for: Nonalcoholic fatty liver disease with elevated alanine aminotransferase levels is negatively associated with bone mineral density: Cross-sectional study in U.S. adults
Source: PLoS One. 2018 Jun 13;13(6):e0197900. doi: 10.1371/journal.pone.0197900 (PMC5999215; doi:10.1371/journal.pone.0197900)
Supplement: S4 Table — (DOCX) [file pone.0197900.s004.docx]

S4 Table. Characteristics of participants with NAFLD with high or normal ALT levels and participants without NAFLD (n=6015)

|  | HA NAFLD  N=353 | NA NAFLD  N=1263 | Non-NAFLD  N=4399 | P value |
| --- | --- | --- | --- | --- |
| Sum of Sample Weight (%) | 5.3 | 19.1 | 75.7 |  |
| Demographic variables |  |  |  |  |
| Gender (%) |  |  |  | < 0.01 |
| Males | 53.2 | 53.3 | 44.8 |  |
| Premenopausal females | 12.5 | 11.6 | 19.9 |  |
| Postmenopausal females | 34.3 | 35.1 | 35.3 |  |
| Race/Ethnicity (%) |  |  |  | < 0.01 |
| White | 78.7 | 81.6 | 80.0 |  |
| Black | 3.4 | 7.9 | 9.6 |  |
| Mexican-American | 9.9 | 4.6 | 3.0 |  |
| Others | 8.0 | 5.9 | 7.4 |  |
| Age (years) | 52.6 | 56.4 | 54.0 | < 0.01 |
| Body Weight (kg) ^1^ | 93.0 | 86.1 | 74.3 | < 0.01 |
| BMI (kg/m2) ^1^ | 32.6 | 30.2 | 26.4 | < 0.01 |
| Clinical variables |  |  |  |  |
| AST (U/L) ^2^ | 36.4 | 20.0 | 20.0 | < 0.01 |
| ALT (U/L) ^2^ | 43.4 | 16.4 | 15.2 | < 0.01 |
| Bone mineral density |  |  |  |  |
| Femoral neck (g/cm2) | 0.82 | 0.80 | 0.77 | < 0.01 |
| Osteoporosis (%) | 2.8 | 3.9 | 5.6 | 0.06 |
| Osteopenia (%) | 33.3 | 33.8 | 42.8 | < 0.01 |
| Detailed Clinical Variables |  |  |  |  |
| Creatinine (mg/dl) ^2^ | 0.87 | 0.87 | 0.85 | 0.03 |
| Estimated GFR (ml/min) ^2^ | 85.1 | 84.4 | 85.7 | 0.22 |
| Calcium (mmol/L) ^3^ | 1.23 | 1.23 | 1.23 | 0.42 |
| Thyroid Stimulating Hormone(TSH) (U/ml) ^4^ | 3.60 | 2.44 | 2.32 | 0.38 |
| Platelet (10^4/μL) ^5^ | 26.4 | 27.5 | 27.0 | 0.24 |
| 25(OH) Vitamin D (ng/ml) ^6^ | 70.7 | 69.5 | 72.0 | 0.31 |
| HOMA-IR ^7^ | 6.09 | 3.92 | 2.20 | < 0.01 |

Abbreviations: AST, Aspartate Aminotransferase; ALT, Alanine Aminotransferase; HA NAFLD, NAFLD with high alanine aminotransferase levels; NA NAFLD, NAFLD with normal alanine aminotransferase levels

Values are shown as weighted mean or weighted percentage. The HA NAFLD group included participants with moderate or severe steatosis with high ALT levels, the NA NAFLD group included participants with moderate or severe steatosis with normal ALT levels, and the non-NAFLD group included participants with mild steatosis or normal liver. 1 n=6006, 2 n=5800, 3 n=5334, 4 n=5722, 5 n=5853, 6 n=5886, 7 n=5008
