# Supplementary material for: Nonalcoholic fatty liver disease with elevated alanine aminotransferase levels is negatively associated with bone mineral density: Cross-sectional study in U.S. adults
Source: PLoS One. 2018 Jun 13;13(6):e0197900. doi: 10.1371/journal.pone.0197900 (PMC5999215; doi:10.1371/journal.pone.0197900)
Supplement: S5 Table — (DOCX) [file pone.0197900.s005.docx]

S5 Table. Femoral neck BMD for participants with NAFLD with high or normal alanine aminotransferase (ALT) levels and participants without NAFLD, stratified by gender and menopausal status, race/ethnicity, age, and BMI

|  | HA NAFLD | NA NAFLD | Non-NAFLD | P value |
| --- | --- | --- | --- | --- |
| Gender and menopausal status |  |  |  |  |
| Males | 0.845 (0.016) | 0.838 (0.007) | 0.813 (0.004) | < 0.01 |
| Premenopausal Females | 0.835 (0.031) | 0.823 (0.011) | 0.804 (0.007) | 0.20 |
| Postmenopausal Females | 0.762 (0.021) | 0.733 (0.007) | 0.701 (0.005) | < 0.01 |
| Race/Ethnicity |  |  |  |  |
| White | 0.806 (0.012) | 0.790 (0.006) | 0.760 (0.004) | < 0.01 |
| Black | 0.869 (0.030) | 0.902 (0.010) | 0.868 (0.006) | 0.01 |
| Mexican American | 0.847 (0.010) | 0.825 (0.007) | 0.813 (0.005) | < 0.01 |
| Age |  |  |  |  |
| 40 – 50 years | 0.851 (0.020) | 0.869 (0.010) | 0.819 (0.005) | < 0.01 |
| 50 – 60 years | 0.800 (0.020) | 0.802 (0.010) | 0.771 (0.006) | 0.02 |
| 60 – 75 years | 0.776 (0.021) | 0.749 (0.007) | 0.712 (0.004) | < 0.01 |
| BMI |  |  |  |  |
| - 25 kg/cm2 | 0.684 (0.041) | 0.713 (0.013) | 0.725 (0.005) | 0.47 |
| 25 - 30 kg/cm2 | 0.756 (0.020) | 0.785 (0.009) | 0.788 (0.004) | 0.22 |
| 30 - 35 kg/cm2 | 0.824 (0.016) | 0.821 (0.010) | 0.827 (0.009) | 0.89 |
| 35 - kg /cm2 | 0.893 (0.018) | 0.869 (0.014) | 0.878 (0.013) | 0.59 |

Abbreviations: AST, Aspartate Aminotransferase; ALT, Alanine Aminotransferase; HA NAFLD, NAFLD with high alanine aminotransferase levels; NA NAFLD, NAFLD with normal alanine aminotransferase levels

Data are expressed as estimates (standard error).

The HA NAFLD group included participants with moderate or severe steatosis with high ALT levels, the NA NAFLD group included participants with moderate or severe steatosis with normal ALT levels, and the non-NAFLD group included participants with mild steatosis or normal liver.
