# Supplementary material for: Nonalcoholic fatty liver disease with elevated alanine aminotransferase levels is negatively associated with bone mineral density: Cross-sectional study in U.S. adults
Source: PLoS One. 2018 Jun 13;13(6):e0197900. doi: 10.1371/journal.pone.0197900 (PMC5999215; doi:10.1371/journal.pone.0197900)
Supplement: S6 Table — (DOCX) [file pone.0197900.s006.docx]

S6 Table. Secondary multiple linear regression analysis assessing the effects of NAFLD with high or normal alanine aminotransferase (ALT) levels on bone mineral density (N=5751)

|  | Full Model  (Secondary Analysis) | P value | Model 2 | P value |
| --- | --- | --- | --- | --- |
| Intercept | 0.811 (0.004) | < 0.01 | 0.812 (0.004) | < 0.01 |
| High ALT (HA) NAFLD | −0.0414 (0.0184) | 0.03 | −0.0230 (0.0104) | 0.03 |
| Normal ALT (NA) NAFLD | 0.0071 (0.0087) | 0.42 | −0.0027 (0.0053) | 0.62 |
| Non-NAFLD | Ref. |  | Ref. |  |
| Postmenopausal | −0.098 (0.005) | < 0.01 | −0.100 (0.005) | < 0.01 |
| Premenopausal | −0.036 (0.007) | < 0.01 | −0.039 (0.006) | < 0.01 |
| Male | Ref. |  | Ref. |  |
| Black | 0.088 (0.005) | < 0.01 | 0.089 (0.005) | < 0.01 |
| Mexican-American | 0.027 (0.005) | < 0.01 | 0.026 (0.004) | < 0.01 |
| White | Ref. |  | Ref. |  |
| Age | −0.0034 (0.0002) | < 0.01 | −0.0034 (0.0002) | < 0.01 |
| BMI | 0.0098 (0.0007) | < 0.01 | 0.0097 (0.0005) | < 0.01 |
| Interaction terms |  |  |  |  |
| HA/NA NAFLD * gender/menopausal |  | 0.44 |  |  |
| HA NAFLD * Postmenopausal | −0.006 (0.024) | 0.79 |  |  |
| HA NAFLD * Premenopausal | 0.027 (0.031) | 0.40 |  |  |
| NA NAFLD * Postmenopausal | −0.008 (0.012) | 0.50 |  |  |
| NA NAFLD * Premenopausal | −0.027 (0.017) | 0.12 |  |  |
| HA/NA NAFLD * races |  | 0.70 |  |  |
| HA NAFLD * Black | −0.008 (0.029) | 0.79 |  |  |
| HA NAFLD * Mexican-American | 0.015 (0.019) | 0.43 |  |  |
| NA NAFLD * Black | 0.007 (0.011) | 0.50 |  |  |
| NA NAFLD * Mexican-American | −0.007 (0.011) | 0.57 |  |  |
| HA/NA NAFLD * age |  | 0.31 |  |  |
| HA NAFLD * Age | 0.0018 (0.0012) | 0.13 |  |  |
| NA NAFLD * Age | −0.0003 (0.0007) | 0.65 |  |  |
| HA/NA NAFLD * BMI |  | 0.05 |  |  |
| HA NAFLD * BMI | 0.003 (0.002) | 0.08 |  |  |
| NA NAFLD * BMI | −0.001 (0.001) | 0.30 |  |  |

Data are expressed as beta estimates (standard error). The HA NAFLD group included participants with moderate or severe steatosis with high ALT levels, the NA NAFLD group included participants with moderate or severe steatosis with normal ALT levels, and the non-NAFLD group included participants with mild steatosis or normal liver. Only Black, Mexican-American, and White participants were used in this analysis, and participants with other race/ethnicities were not used (described in method section). Age and BMI were dealt as continuous variables. Age variable and BMI variable were centered in these models around overall means, 54 and 27 respectively.

Abbreviations: HA NAFLD, NAFLD with high alanine aminotransferase levels; NA NAFLD, NAFLD with normal alanine aminotransferase levels.

Full model (in the secondary analysis) had gender and menopausal status, race/ethnicity, age, BMI, and their interactions with NAFLD status as covariates. Interaction terms in the full model were assessed, and insignificant terms were removed iteratively using backward elimination. As a result, all the interaction terms were not included in the final model, Model 2.
