# Supplementary material for: Nonalcoholic fatty liver disease with elevated alanine aminotransferase levels is negatively associated with bone mineral density: Cross-sectional study in U.S. adults
Source: PLoS One. 2018 Jun 13;13(6):e0197900. doi: 10.1371/journal.pone.0197900 (PMC5999215; doi:10.1371/journal.pone.0197900)
Supplement: S7 Table — Secondary multiple linear regression analysis assessing the effects of NAFLD with high or normal alanine aminotransferase (ALT) levels on bone mineral density for each gender and menopausal status (A) Males (B) Premenopausal Females (C) Postmenopausal Females. (DOCX) [file pone.0197900.s007.docx]

S7 Table. Secondary multiple linear regression analysis assessing the effects of NAFLD with high or normal alanine aminotransferase (ALT) levels on bone mineral density for each gender and menopausal status (A) Males (n=2707) (B) Premenopausal Females (n=849) (C) Postmenopausal Females (n=2195)

(A)

|  | Full Model  (Secondary Analysis) | P value | Model 2A | P value |
| --- | --- | --- | --- | --- |
| Intercept | 0.811 (0.004) | < 0.01 | 0.811 (0.004) | < 0.01 |
| High ALT (HA) NAFLD | −0.0654 (0.0193) | < 0.01 | −0.0597 (0.0164) | < 0.01 |
| Normal ALT (NA) NAFLD | 0.0034 (0.0101) | 0.74 | 0.0006 (0.0080) | 0.94 |
| Non-NAFLD | Ref. |  | Ref. |  |
| Black | 0.093 (0.008) | < 0.01 | 0.093 (0.007) | < 0.01 |
| Mexican-American | 0.032 (0.008) | < 0.01 | 0.034 (0.007) | < 0.01 |
| White | Ref. |  | Ref. |  |
| Age | −0.0025 (0.0004) | < 0.01 | −0.0026 (0.0003) | < 0.01 |
| BMI | 0.0120 (0.0010) | < 0.01 | 0.0120 (0.0010) | < 0.01 |
| Interaction terms |  |  |  |  |
| HA/NA NAFLD * races |  | 0.36 |  |  |
| HA NAFLD * Black | 0.052 (0.041) | 0.22 |  |  |
| HA NAFLD * Mexican-American | 0.041 (0.024) | 0.10 |  |  |
| NA NAFLD * Black | −0.043 (0.015) | 0.77 |  |  |
| NA NAFLD * Mexican-American | −0.012 (0.015) | 0.46 |  |  |
| HA/NA NAFLD * age |  | 0.23 |  |  |
| HA NAFLD * Age | 0.0019 (0.0014) | 0.18 |  |  |
| NA NAFLD * Age | −0.0009 (0.0009) | 0.35 |  |  |
| HA/NA NAFLD * BMI |  | 0.01 |  | 0.04 |
| HA NAFLD * BMI | 0.005 (0.002) | 0.03 | 0.004 (0.002) | 0.06 |
| NA NAFLD * BMI | −0.002 (0.002) | 0.24 | −0.002 (0.002) | 0.31 |

(B)

|  | Full Model  (Secondary Analysis) | P value | Model 2B | P value |
| --- | --- | --- | --- | --- |
| Intercept | 0.771 (0.012) | < 0.01 | 0.765 (0.011) | < 0.01 |
| High ALT (HA) NAFLD | −0.0337 (0.0391) | 0.39 | −0.0078 (0.0319) | 0.81 |
| Normal ALT (NA) NAFLD | −0.0441 (0.0256) | 0.09 | −0.0181 (0.0166) | 0.28 |
| Non-NAFLD | Ref. |  | Ref. |  |
| Black | 0.088 (0.014) | < 0.01 | 0.094 (0.012) | < 0.01 |
| Mexican-American | 0.028 (0.013) | 0.04 | 0.027 (0.013) | 0.04 |
| White | Ref. |  | Ref. |  |
| Age | −0.0038 (0.0012) | < 0.01 | −0.0044 (0.0011) | < 0.01 |
| BMI | 0.0097 (0.0016) | < 0.01 | 0.0094 (0.0012) | < 0.01 |
| Interaction terms |  |  |  |  |
| HA/NA NAFLD * races |  | 0.45 |  |  |
| HA NAFLD * Black | −0.017 (0.046) | 0.72 |  |  |
| HA NAFLD * Mexican-American | −0.046 (0.052) | 0.39 |  |  |
| NA NAFLD * Black | 0.051 (0.030) | 0.09 |  |  |
| NA NAFLD * Mexican-American | 0.014 (0.026) | 0.59 |  |  |
| HA/NA NAFLD * age |  | 0.51 |  |  |
| HA NAFLD * Age | −0.0019 (0.0048) | 0.70 |  |  |
| NA NAFLD * Age | −0.0028 (0.0024) | 0.25 |  |  |
| HA/NA NAFLD * BMI |  | 0.46 |  |  |
| HA NAFLD * BMI | 0.005 (0.005) | 0.38 |  |  |
| NA NAFLD * BMI | −0.001 (0.002) | 0.43 |  |  |

(C)

|  | Full Model  (Secondary Analysis) | P value | Model 2C | P value |
| --- | --- | --- | --- | --- |
| Intercept | 0.721 (0.005) | < 0.01 | 0.720 (0.005) | < 0.01 |
| High ALT (HA) NAFLD | −0.0307 (0.0304) | 0.32 | −0.0152 (0.0177) | 0.40 |
| Normal ALT (NA) NAFLD | −0.0028 (0.0161) | 0.86 | 0.0020 (0.0079) | 0.80 |
| Non-NAFLD | Ref. |  | Ref. |  |
| Black | 0.086 (0.007) | < 0.01 | 0.085 (0.007) | < 0.01 |
| Mexican-American | 0.020 (0.008) | 0.02 | 0.017 (0.006) | < 0.01 |
| White | Ref. |  | Ref. |  |
| Age | −0.0050 (0.0004) | < 0.01 | −0.0047 (0.0004) | < 0.01 |
| BMI | 0.0082 (0.0006) | < 0.01 | 0.0083 (0.0005) | < 0.01 |
| Interaction terms |  |  |  |  |
| HA/NA NAFLD * races |  | 0.65 |  |  |
| HA NAFLD * Black | −0.060 (0.040) | 0.14 |  |  |
| HA NAFLD * Mexican-American | −0.001 (0.032) | 0.97 |  |  |
| NA NAFLD * Black | 0.0004 (0.0178) | 0.98 |  |  |
| NA NAFLD * Mexican-American | −0.009 (0.020) | 0.66 |  |  |
| HA/NA NAFLD * age |  | 0.36 |  |  |
| HA NAFLD * Age | 0.0028 (0.0021) | 0.19 |  |  |
| NA NAFLD * Age | 0.0008 (0.0013) | 0.53 |  |  |
| HA/NA NAFLD * BMI |  | 0.74 |  |  |
| HA NAFLD * BMI | 0.0017 (0.0023) | 0.45 |  |  |
| NA NAFLD * BMI | −0.0000 (0.0011) | 0.99 |  |  |

Multiple linear regression analysis was conducted for each gender and menopausal status (A) Males (B) Premenopausal Females (C) Postmenopausal Females.

Data are expressed as beta estimates (standard error). The HA NAFLD group included participants with moderate or severe steatosis with high ALT levels, the NA NAFLD group included participants with moderate or severe steatosis with normal ALT levels, and the non-NAFLD group included participants with mild steatosis or normal liver. Only Black, Mexican-American, and White participants were used in this analysis, and participants with other race/ethnicities were not used (described in method section). Age and BMI were dealt as continuous variables. Age variable and BMI variable were centered in these models around overall means, 54 and 27 respectively.

Full model had gender and menopausal status, race/ethnicity, age, BMI, and their interactions with NAFLD status as covariates. Interaction terms in the full model were assessed, and insignificant terms were removed iteratively using backward elimination. Model 2A-C are the final model derived from the full models. Only among males, the interaction term between NAFLD and BMI remained (Model 2A).
