# Supplementary material for: Nonalcoholic fatty liver disease with elevated alanine aminotransferase levels is negatively associated with bone mineral density: Cross-sectional study in U.S. adults
Source: PLoS One. 2018 Jun 13;13(6):e0197900. doi: 10.1371/journal.pone.0197900 (PMC5999215; doi:10.1371/journal.pone.0197900)
Supplement: S8 Table — (DOCX) [file pone.0197900.s008.docx]

S8 Table. Mean values of HOMA-IR for the NAFLD groups for different levels of BMI (n=4643)

|  | HA NAFLD  (n=267) | NA NAFLD  (n=944) | Non-NAFLD  (n=3432) |
| --- | --- | --- | --- |
| BMI |  |  |  |
| 15-20 | No observations | 1.35 (0.21) | 1.25 (0.06) |
| 20-25 | 2.09 (0.49) | 1.99 (0.12) | 1.57 (0.04) |
| 25-30 | 4.09 (0.34) | 3.24 (0.12) | 2.25 (0.06) |
| 30-35 | 6.02 (0.66) | 4.38 (0.51) | 3.12 (0.12) |
| 35-40 | 8.26 (1.53) | 5.86 (0.89) | 4.71 (0.63) |

Abbreviation: HOMA, homeostatic model assessment; IR, insulin resistance; HA NAFLD, NAFLD with high alanine aminotransferase levels; NA NAFLD, NAFLD with normal alanine aminotransferase levels.

Data are expressed as mean estimates (standard error). HOMA-IR quantifies the strength of insulin resistance. HOMA-IR was calculated by serum insulin (μU/mL) * serum glucose (mmol/L) / 22.5. Serum insulin and serum glucose should be measured at fasting state. I only included people who ate or drank last more than 6 hours before. To exclude extremely low or high blood glucose people, only the people with glucose level of 50mg/dl to 250mg/dl were included.
