# Supplementary material for: Nonalcoholic fatty liver disease with elevated alanine aminotransferase levels is negatively associated with bone mineral density: Cross-sectional study in U.S. adults
Source: PLoS One. 2018 Jun 13;13(6):e0197900. doi: 10.1371/journal.pone.0197900 (PMC5999215; doi:10.1371/journal.pone.0197900)
Supplement: S9 Table — (DOCX) [file pone.0197900.s009.docx]

S9 Table. Mean values of HOMA-IR for the NAFLD groups for different levels of BMI among males (n=2219)

|  | HA NAFLD  (n=134) | NA NAFLD  (n=489) | Non-NAFLD  (n=1596) |
| --- | --- | --- | --- |
| BMI |  |  |  |
| 15-20 | No observations | 0.89 (0.11) | 1.29 (0.06) |
| 20-25 | 2.07 (0.20) | 2.04 (0.20) | 1.62 (0.06) |
| 25-30 | 4.06 (0.61) | 3.07 (0.14) | 2.31 (0.08) |
| 30-35 | 5.88 (0.87) | 4.85 (0.76) | 3.48 (0.19) |
| 35-40 | 12.34 (3.46) | 8.42 (2.64) | 5.47 (0.99) |

Abbreviation: HOMA, homeostatic model assessment; IR, insulin resistance; HA NAFLD, NAFLD with high alanine aminotransferase levels; NA NAFLD, NAFLD with normal alanine aminotransferase levels.

Data are expressed as mean estimates (standard error). HOMA-IR quantifies the strength of insulin resistance. HOMA-IR was calculated by serum insulin (uU/mL) * serum glucose (mmol/L) / 22.5. Serum insulin and serum glucose should be measured at fasting state. I only included people who ate or drank last more than 6 hours before. To exclude extremely low or high blood glucose people, only the people with glucose level of 50mg/dl to 250mg/dl were included.
