# Supplementary material for: Nonalcoholic fatty liver disease with elevated alanine aminotransferase levels is negatively associated with bone mineral density: Cross-sectional study in U.S. adults
Source: PLoS One. 2018 Jun 13;13(6):e0197900. doi: 10.1371/journal.pone.0197900 (PMC5999215; doi:10.1371/journal.pone.0197900)
Supplement: S10 Table — (DOCX) [file pone.0197900.s010.docx]

S10 Table. Mean values of serum vitamin D (25 OH D) for the NAFLD groups for different levels of BMI (n=5433)

|  | HA NAFLD  (n=317) | NA NAFLD  (n=1126) | Non-NAFLD  (n=3990) |
| --- | --- | --- | --- |
| BMI |  |  |  |
| 15-20 | No observations | 75.07 (5.95) | 65.58 (2.74) |
| 20-25 | 64.78 (5.99) | 75.72 (3.28) | 77.25 (1.32) |
| 25-30 | 74.24 (4.39) | 73.52 (2.13) | 72.58 (1.12) |
| 30-35 | 72.03 (5.70) | 68.86 (2.56) | 69.44 (2.37) |
| 35-40 | 73.47 (8.02) | 57.86 (2.05) | 60.55 (2.52) |

Abbreviation: HA NAFLD, NAFLD with high alanine aminotransferase levels; NA NAFLD, NAFLD with normal alanine aminotransferase levels.

Data are expressed as mean estimates (standard error) for vitamin D (nmol/L).
