# Supplementary material for: Nonalcoholic fatty liver disease with elevated alanine aminotransferase levels is negatively associated with bone mineral density: Cross-sectional study in U.S. adults
Source: PLoS One. 2018 Jun 13;13(6):e0197900. doi: 10.1371/journal.pone.0197900 (PMC5999215; doi:10.1371/journal.pone.0197900)
Supplement: S11 Table — (DOCX) [file pone.0197900.s011.docx]

S11 Table. Mean values of serum vitamin D (25 OH D) for the NAFLD groups for different levels of BMI among males (n=2622)

|  | HA NAFLD  (n=165) | NA NAFLD  (n=584) | Non-NAFLD  (n=1873) |
| --- | --- | --- | --- |
| BMI |  |  |  |
| 15-20 | No observations | 72.58 (9.16) | 67.71 (5.04) |
| 20-25 | 72.89 (7.56) | 75.84 (5.04) | 82.39 (2.04) |
| 25-30 | 73.46 (4.67) | 78.47 (2.88) | 77.60 (1.51) |
| 30-35 | 80.78 (6.52) | 73.02 (3.11) | 77.41 (2.67) |
| 35-40 | 90.80 (10.57) | 63.60 (5.63) | 67.63 (4.01) |

Abbreviation: HA NAFLD, NAFLD with high alanine aminotransferase levels; NA NAFLD, NAFLD with normal alanine aminotransferase levels.

Data are expressed as mean estimates (standard error) for vitamin D (nmol/L).
