# Supplementary material for: Nonalcoholic fatty liver disease with elevated alanine aminotransferase levels is negatively associated with bone mineral density: Cross-sectional study in U.S. adults
Source: PLoS One. 2018 Jun 13;13(6):e0197900. doi: 10.1371/journal.pone.0197900 (PMC5999215; doi:10.1371/journal.pone.0197900)
Supplement: S1 Fig — The association assessed in this study is confounded by gender and menopausal status, age, BMI, and races. (DOCX) [file pone.0197900.s012.docx]

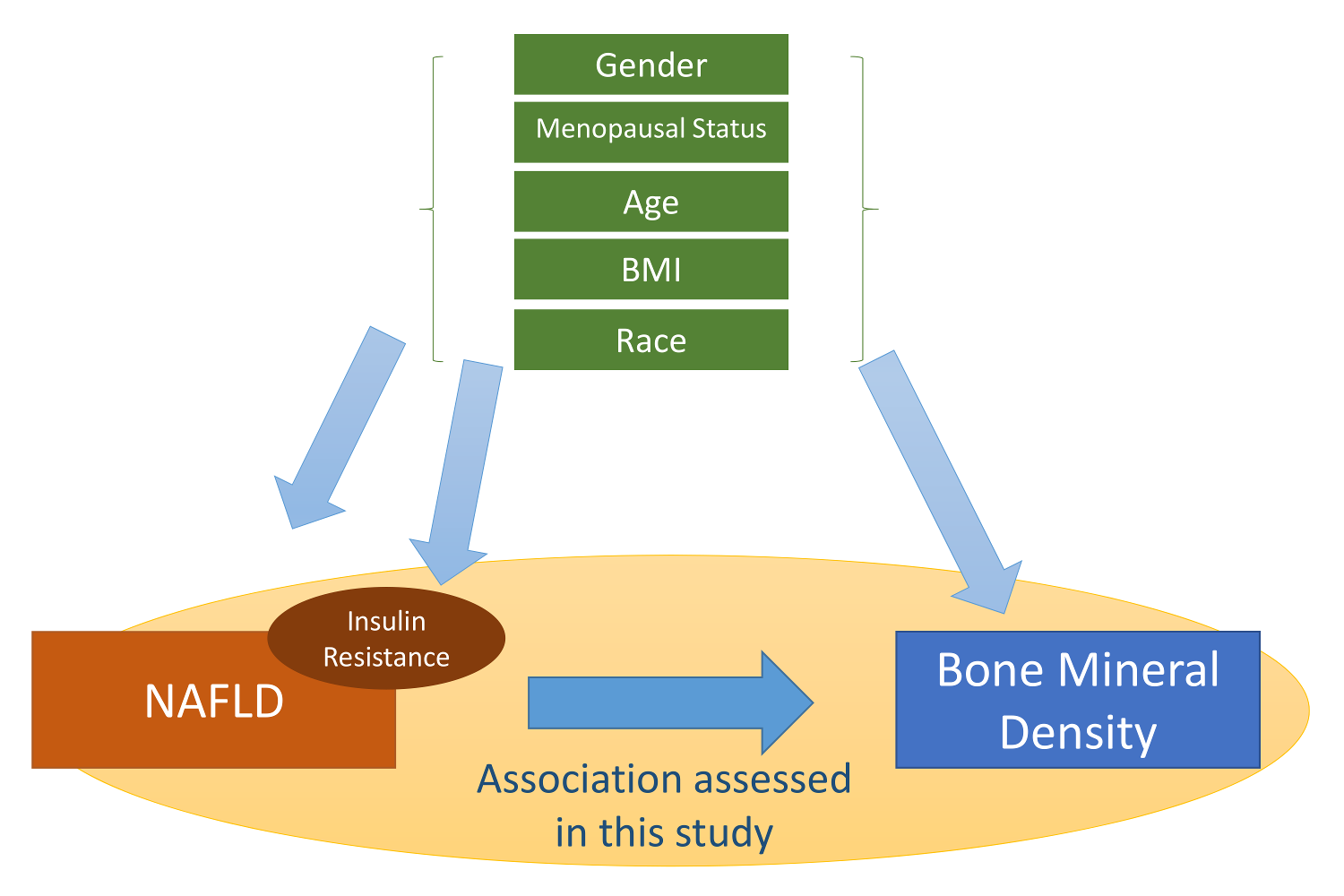


S1 Fig. The association assessed in this study is confounded by gender and menopausal status, age, BMI, and races.
