# Supplementary material for: Nonalcoholic fatty liver disease with elevated alanine aminotransferase levels is negatively associated with bone mineral density: Cross-sectional study in U.S. adults
Source: PLoS One. 2018 Jun 13;13(6):e0197900. doi: 10.1371/journal.pone.0197900 (PMC5999215; doi:10.1371/journal.pone.0197900)
Supplement: S2 Fig — (DOCX) [file pone.0197900.s013.docx]

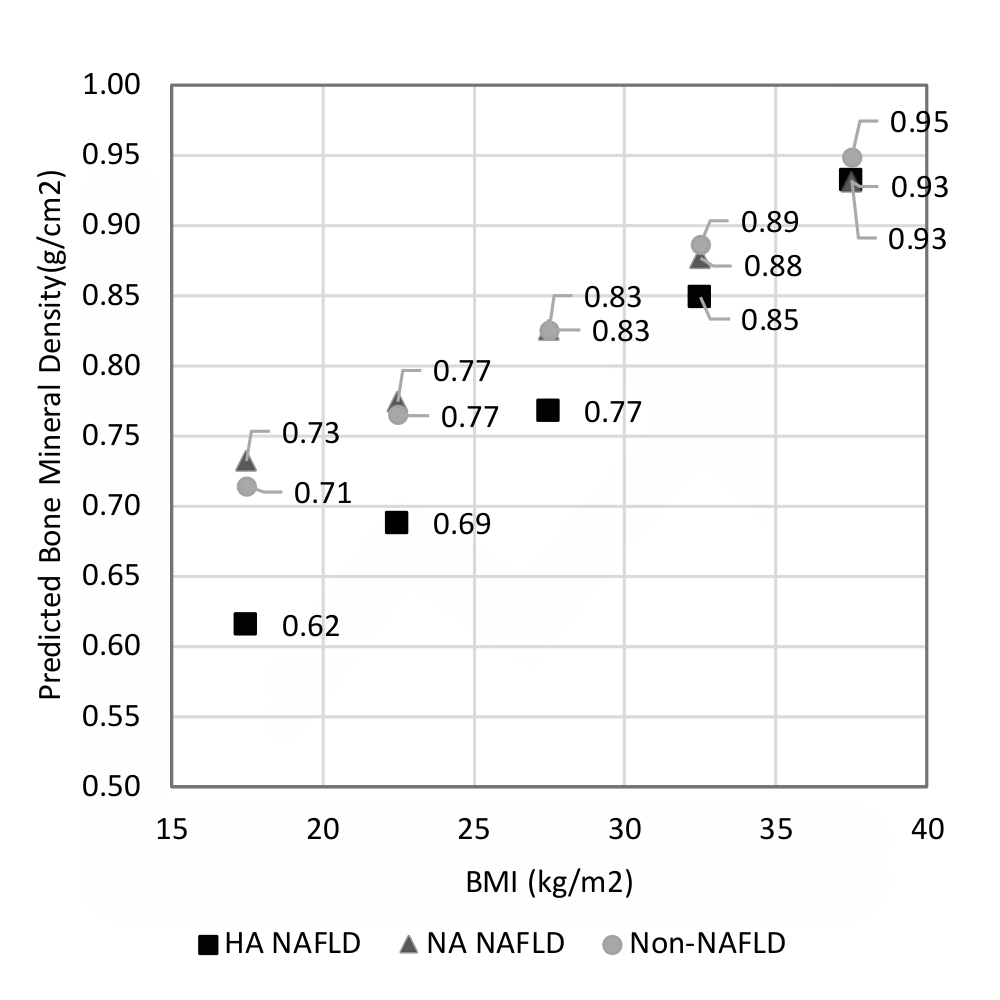


S2 Fig. Predicted femoral neck bone mineral densities for the NAFLD groups for different levels of BMI among males

Predictions were made using the final model of males, model 2A. For covariate variables, representative values were used for each group in the same way as done for Figure 2 (details described in method section). Abbreviations: HA NAFLD, NAFLD with high alanine aminotransferase levels; NA NAFLD, NAFLD with normal alanine aminotransferase levels.
