# Supplementary material for: Nonalcoholic fatty liver disease with elevated alanine aminotransferase levels is negatively associated with bone mineral density: Cross-sectional study in U.S. adults
Source: PLoS One. 2018 Jun 13;13(6):e0197900. doi: 10.1371/journal.pone.0197900 (PMC5999215; doi:10.1371/journal.pone.0197900)
Supplement: S3 Fig — (DOCX) [file pone.0197900.s014.docx]

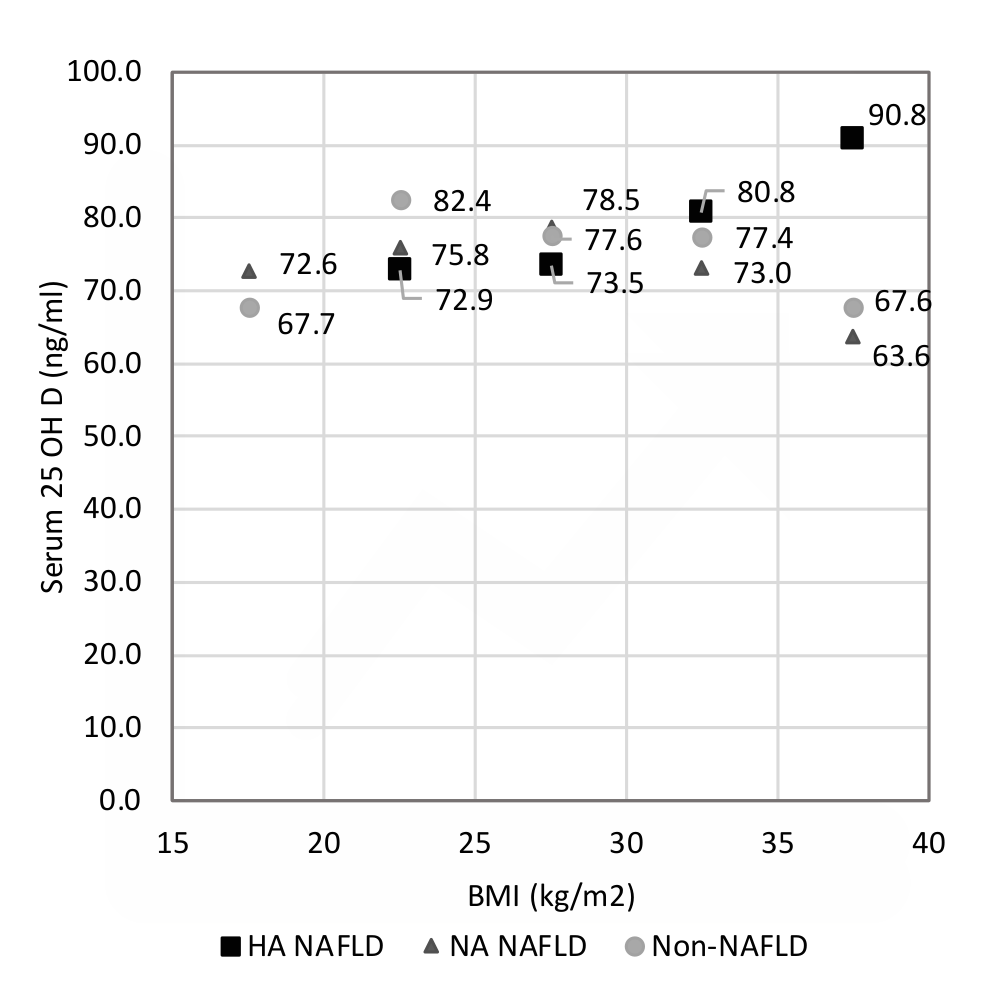


S3 Fig. 25(OH)D levels for the NAFLD groups for different levels of BMI among males

The male population was stratified by BMI into the ranges, 15-20, 20-25, 25-30, 30-35, and 35-40 kg/m2 groups. No one in the HA NAFLD group had a BMI in the range of 15-20 kg/m2. Mean 25(OH)D values were obtained for each NAFLD group for different levels of BMI. Abbreviations: 25(OH)D, 25-hydroxyvitamin D; HA NAFLD, NAFLD with high alanine aminotransferase levels; NA NAFLD, NAFLD with normal alanine aminotransferase levels.
