# Supplementary material for: Nonalcoholic fatty liver disease with elevated alanine aminotransferase levels is negatively associated with bone mineral density: Cross-sectional study in U.S. adults
Source: PLoS One. 2018 Jun 13;13(6):e0197900. doi: 10.1371/journal.pone.0197900 (PMC5999215; doi:10.1371/journal.pone.0197900)
Supplement: S1 File — (DOC) [file pone.0197900.s015.doc]

STROBE Statement—Checklist of items that should be included in reports of ***cross-sectional studies***

|  | Item No | Recommendation |
| --- | --- | --- |
| **Title and abstract** | 1 | (*a*) Indicate the study’s design with a commonly used term in the title or the abstract  **Described in title.** |
|  |  | (*b*) Provide in the abstract an informative and balanced summary of what was done and what was found  **Described in abstract.** |
| Introduction | | |
| Background/rationale | 2 | Explain the scientific background and rationale for the investigation being reported  **Described in introduction. Previous literatures are reviewed.** |
| Objectives | 3 | State specific objectives, including any prespecified hypotheses  **Described in introduction.** |
| Methods | | |
| Study design | 4 | Present key elements of study design early in the paper  **Described in Methods. Cross-sectional analysis was conducted.** |
| Setting | 5 | Describe the setting, locations, and relevant dates, including periods of recruitment, exposure, follow-up, and data collection  **Described in Methods**. |
| Participants | 6 | (*a*) Give the eligibility criteria, and the sources and methods of selection of participants  **Described in Methods** |
| Variables | 7 | Clearly define all outcomes, exposures, predictors, potential confounders, and effect modifiers. Give diagnostic criteria, if applicable  **Described in Methods** |
| Data sources/ measurement | 8* | For each variable of interest, give sources of data and details of methods of assessment (measurement). Describe comparability of assessment methods if there is more than one group  **Described in Methods** |
| Bias | 9 | Describe any efforts to address potential sources of bias |
| Study size | 10 | Explain how the study size was arrived at  **Described in Methods** |
| Quantitative variables | 11 | Explain how quantitative variables were handled in the analyses. If applicable, describe which groupings were chosen and why  **Described in Methods** |
| Statistical methods | 12 | (*a*) Describe all statistical methods, including those used to control for confounding  **Described in Methods** |
|  |  | (*b*) Describe any methods used to examine subgroups and interactions  **Described in Methods** |
|  |  | (*c*) Explain how missing data were addressed  **Described in Methods** |
|  |  | (*d*) If applicable, describe analytical methods taking account of sampling strategy  **Described in Methods** |
|  |  | (*e*) Describe any sensitivity analyses |
| Results | | |
| Participants | 13* | (a) Report numbers of individuals at each stage of study—eg numbers potentially eligible, examined for eligibility, confirmed eligible, included in the study, completing follow-up, and analysed  **Described in Methods** |
|  |  | (b) Give reasons for non-participation at each stage  **Described in Methods** |
|  |  | (c) Consider use of a flow diagram  **Described as Fig 1** |
| Descriptive data | 14* | (a) Give characteristics of study participants (eg demographic, clinical, social) and information on exposures and potential confounders  **Described in Results. Table1.** |
|  |  | (b) Indicate number of participants with missing data for each variable of interest  **NA** |
| Outcome data | 15* | Report numbers of outcome events or summary measures  **NA** |
| Main results | 16 | (*a*) Give unadjusted estimates and, if applicable, confounder-adjusted estimates and their precision (eg, 95% confidence interval). Make clear which confounders were adjusted for and why they were included  **Described in Results.** |
|  |  | (*b*) Report category boundaries when continuous variables were categorized  **Described in Methods** |
|  |  | (*c*) If relevant, consider translating estimates of relative risk into absolute risk for a meaningful time period  **NA** |
| Other analyses | 17 | Report other analyses done—eg analyses of subgroups and interactions, and sensitivity analyses  **Secondary analysis in Results. Additional analysis for each BMI group was described in Discussion.** |
| Discussion | | |
| Key results | 18 | Summarise key results with reference to study objectives  **Summarized in the 1st paragraph in Discussion.** |
| Limitations | 19 | Discuss limitations of the study, taking into account sources of potential bias or imprecision. Discuss both direction and magnitude of any potential bias  **Described in discussion** |
| Interpretation | 20 | Give a cautious overall interpretation of results considering objectives, limitations, multiplicity of analyses, results from similar studies, and other relevant evidence  **Described in discussion** |
| Generalisability | 21 | Discuss the generalisability (external validity) of the study results  **Described in discussion** |
| Other information | | |
| Funding | 22 | Give the source of funding and the role of the funders for the present study and, if applicable, for the original study on which the present article is based  **Submitted online.** |

*Give information separately for exposed and unexposed groups.

**Note:** An Explanation and Elaboration article discusses each checklist item and gives methodological background and published examples of transparent reporting. The STROBE checklist is best used in conjunction with this article (freely available on the Web sites of PLoS Medicine at http://www.plosmedicine.org/, Annals of Internal Medicine at http://www.annals.org/, and Epidemiology at http://www.epidem.com/). Information on the STROBE Initiative is available at www.strobe-statement.org.
